# Averting Unnecessary Radical Surgery: Xanthogranulomatous Cholecystitis Mimicking Gallbladder Carcinoma

**DOI:** 10.7759/cureus.115461

**Published:** 2026-08-30

**Authors:** Syamasis Bandyopadhyay, Sourav Pratihar, Sandip K Chandra, Aheli Ghosh Dastidar, Arghya Sahu

**Affiliations:** 1 Internal Medicine, Apollo Multispeciality Hospitals, Kolkata, IND; 2 Medicine, Apollo Multispeciality Hospitals, Kolkata, IND

**Keywords:** 18f-fluorodeoxyglucose positron emission tomography (18f-fdg pet), carcinoma gallbladder, carcinoma gallbladder mimics, cholecystectomy, chronic cholecystitis, gallbladder wall thickening, xanthogranulomatous cholecystitis

## Abstract

Xanthogranulomatous cholecystitis (XGC) is a rare, benign inflammatory condition that closely mimics gallbladder carcinoma clinically, radiologically, and intraoperatively. We present the case of an elderly Indian man in his 80s with multiple comorbidities, with vomiting and anorexia, whose radiological and positron emission tomography-computed tomography (PET-CT) findings showed a fluorodeoxyglucose (FDG)-avid gallbladder mass with extension into the liver, cystic duct, common hepatic duct, and common bile duct, highly suggestive of gallbladder carcinoma. However, histopathological examination confirmed XGC. Conservative management with follow-up PET-CT imaging showed marked resolution of FDG uptake, supporting the benign inflammatory nature of the disease. This case shows that FDG avidity does not necessarily indicate malignancy and highlights the indispensable role of histopathological confirmation before considering radical surgery. XGC should be considered in the differential diagnosis of gallbladder masses and FDG-avid lesions to avoid misdiagnosis and unnecessary radical surgery, especially in elderly comorbid patients.

## Introduction

Xanthogranulomatous cholecystitis (XGC) is a rare, benign variant of chronic cholecystitis characterized by focal or diffuse destructive inflammation and the presence of lipid-laden macrophages (xanthoma cells or foam cells) and fibrotic tissue within the gallbladder wall. The reported incidence of XGC in cholecystectomy specimens ranges from 1% in the West to 10% in countries such as India, with XGC predominantly affecting individuals over 50 years of age [1]. Although histologically benign, XGC frequently poses a diagnostic challenge as it often mimics gallbladder carcinoma clinically, radiologically, and intraoperatively owing to overlapping features such as right upper quadrant mass and pain, jaundice, nausea, vomiting, anorexia, dense adhesion of the gallbladder to surrounding structures (such as liver, duodenum, and colon), and irregular wall thickening [2]. This may lead to misdiagnosis and unnecessary radical cholecystectomy, which is associated with increased morbidity and mortality [3]. When a suspicious malignant mass is detected in the gallbladder region, XGC must be considered in the differential diagnosis.

Here, we report a case of an elderly man with multiple comorbidities who was found to have an invasive gallbladder mass in both magnetic resonance cholangiopancreatography (MRCP) and positron emission tomography-computed tomography (PET-CT), which was highly suggestive of gallbladder carcinoma. However, endoscopic ultrasound-guided fine-needle biopsy (EUS-FNB) was performed, and histological examination of the obtained tissue demonstrated XGC. Interval imaging after conservative management showed marked regression, highlighting the dynamic inflammatory nature of XGC. Our case exemplifies the diagnostic dilemma where benign inflammatory disease mimics aggressive malignancy. Although advances in cross-sectional imaging, including MRI and FDG PET-CT, have improved the assessment of gallbladder lesions, no single imaging modality can reliably distinguish XGC from gallbladder carcinoma, particularly when extensive inflammatory changes produce apparent local invasion and increased FDG uptake. Recognition of this mimicry is crucial, as accurate preoperative diagnosis can prevent unwarranted radical resections, particularly in elderly comorbid patients.

## Case presentation

An elderly Indian man in his 80s presented with non-bilious vomiting (one to two episodes/day for four to five days) and nausea after meals for a week, associated with anorexia, dyspepsia, generalized weakness, and weight loss of about 3 kg over a month. He had multiple comorbidities, including hypertension, hypothyroidism, type 2 diabetes mellitus, and ischemic heart disease, and was on regular medications. There was no history of abdominal pain, fever, or pallor at presentation and no previous surgical history. On clinical examination, he was hemodynamically stable, with a pulse rate of 82/min and blood pressure of 130/80 mm Hg, and was icteric. The abdomen was soft and non-tender, with a negative Murphy’s sign and no palpable mass or organomegaly. The rest of the systemic examination was unremarkable.

Initial laboratory investigations revealed a normal total leukocyte count and normal serum amylase and lipase levels but deranged liver function tests: total bilirubin 9.10 mg/dL, conjugated bilirubin 7.60 mg/dL, alanine aminotransferase (ALT) 276 U/L, aspartate aminotransferase (AST) 206 U/L, and alkaline phosphatase 670 U/L, suggestive of cholestatic injury. Whole-abdomen ultrasonography demonstrated a diffusely thickened gallbladder wall (>10 mm) with cholelithiasis (18 mm) and intrahepatic biliary radicle dilatation. Subsequently, MRCP revealed an ill-defined contracted gallbladder mass with foci of diffusion restriction, hyperintense intramural collections, marked irregular and diffuse thickening apparently involving the liver (segment 5), proximal common bile duct, common hepatic duct, obstruction of the primary biliary confluence (Figure 1, Figure 2), and dilatation of bilobar intrahepatic biliary radicles (Figure 3) suspicious of gallbladder carcinoma.

**Figure 1:**
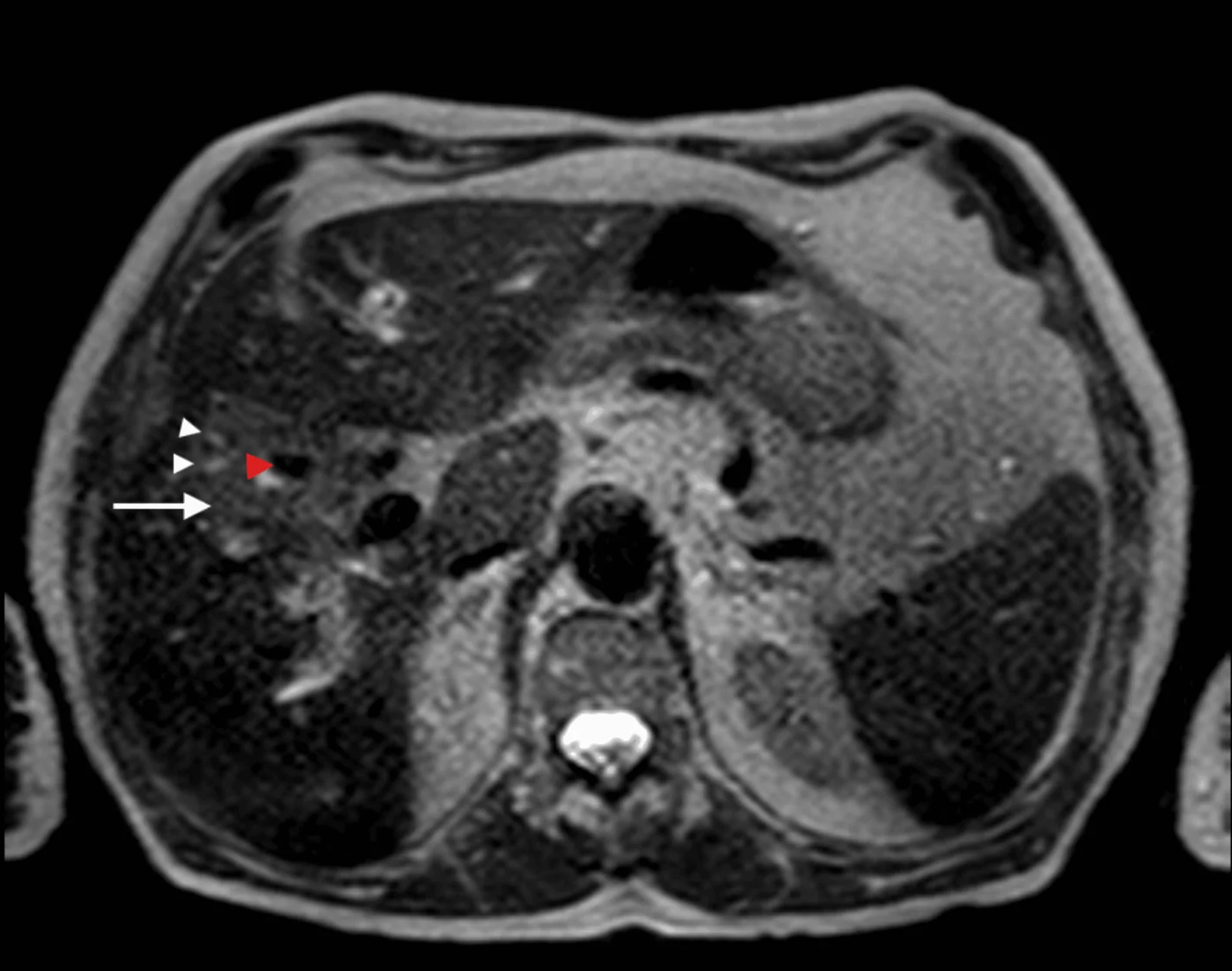
Axial sequential T2W magnetic resonance image showing a contracted gallbladder mass with irregular and diffuse thickening (white arrow), which appears to be infiltrating segment 5 of the liver, calculus (red arrowhead), and hyperintense intramural collections (white arrowheads)

**Figure 2:**
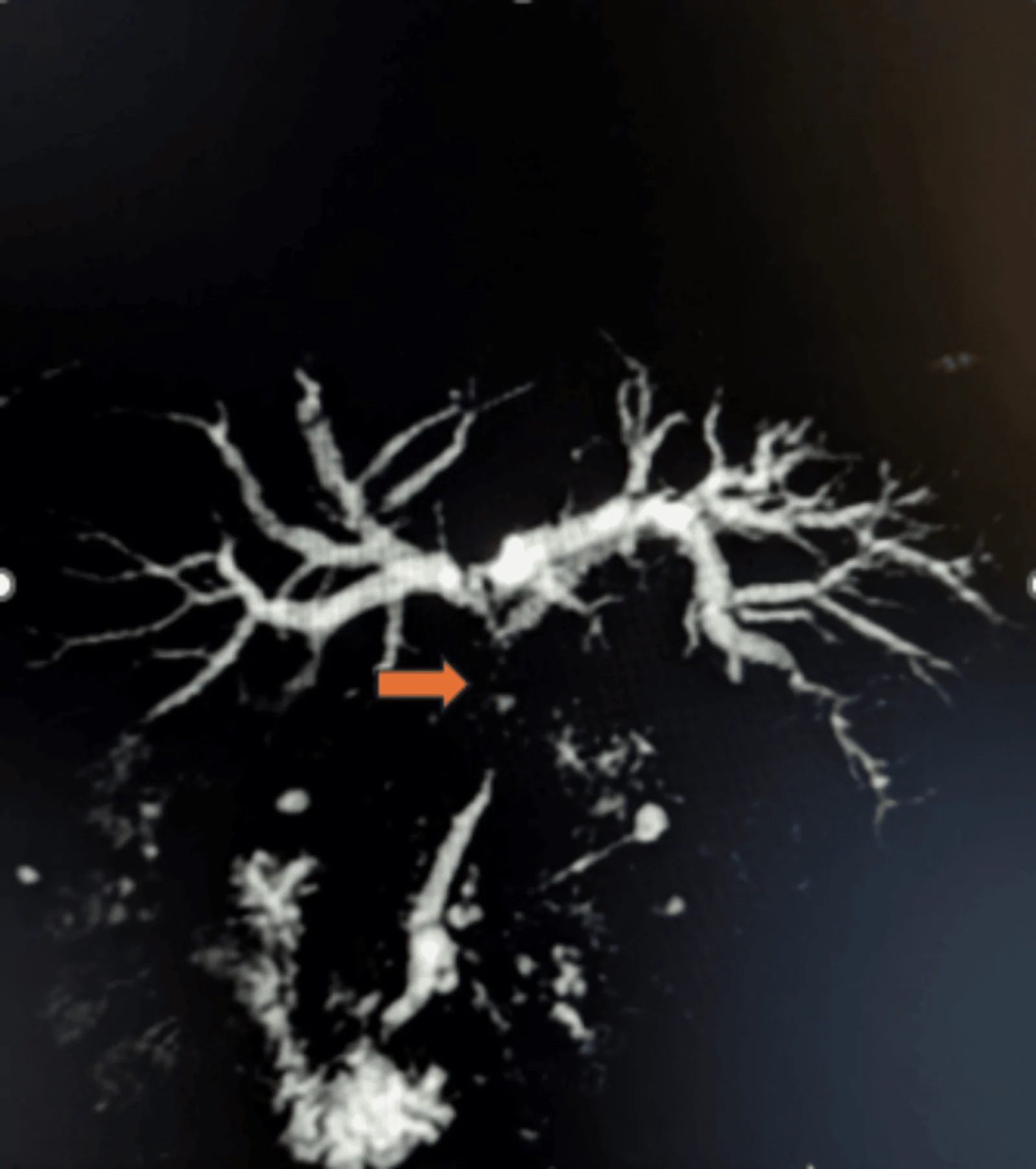
MRCP showing gallbladder mass obstructing proximal common bile duct, common hepatic duct, and primary biliary confluence (orange arrow) MRCP: magnetic resonance cholangiopancreatography

**Figure 3:**
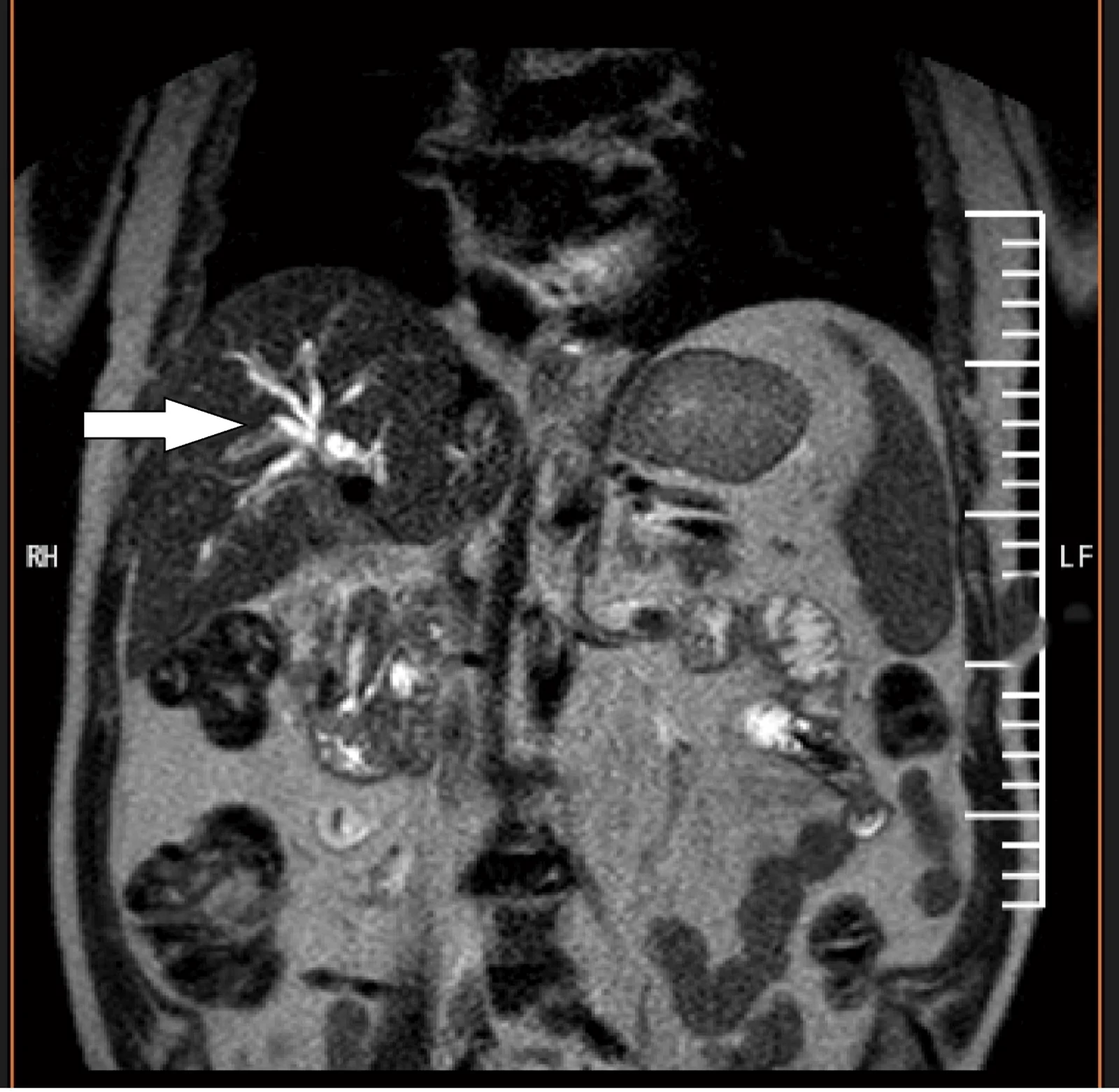
MRCP showing dilatation of intrahepatic biliary radicles (white arrow) MRCP: magnetic resonance cholangiopancreatography, RH: right, LF: left

To further characterize the lesion, fluorine-18 fluorodeoxyglucose (FDG) PET-CT was performed. It revealed a heterogeneously enhancing, FDG-avid mass arising from the gallbladder (42 mm x 31 mm) and extending into the adjacent hepatic tissue, cystic duct, common hepatic duct, and proximal common bile duct. In view of these findings, a provisional diagnosis of gallbladder carcinoma with hepatic invasion was made. Considering the operative risk in an elderly patient and to obtain a definitive tissue diagnosis before performing radical surgery, EUS-FNB from the hypoechoic gallbladder fossa lesion was performed. Histological examination of the EUS-FNB specimen demonstrated features consistent with chronic cholecystitis with xanthogranulomatous inflammation.

Given the benign nature of XGC and the high surgical risk in this elderly patient, the decision for radical cholecystectomy was deferred after multidisciplinary hepatobiliary team review. Cholecystectomy is the definitive treatment for XGC; however, the procedure may be technically challenging because of dense adhesions to surrounding structures, poor visualization of Calot’s triangle, and an increased risk of postoperative complications. The patient declined surgery, and he was managed conservatively with ursodeoxycholic acid tablets (300 mg twice daily) and a short course of antibiotics. After two weeks, endoscopic retrograde cholangiopancreatography-guided placement of a self-expandable metal stent in the common bile duct was performed to relieve obstruction and cholestasis. He was followed up as an outpatient. Follow-up PET-CT after two months demonstrated a significant reduction in FDG uptake (Figure 4) and a smaller gallbladder lesion (30 mm x 22 mm), with normalization of bilirubin and liver enzymes, findings consistent with resolving inflammatory disease rather than malignancy.

**Figure 4:**
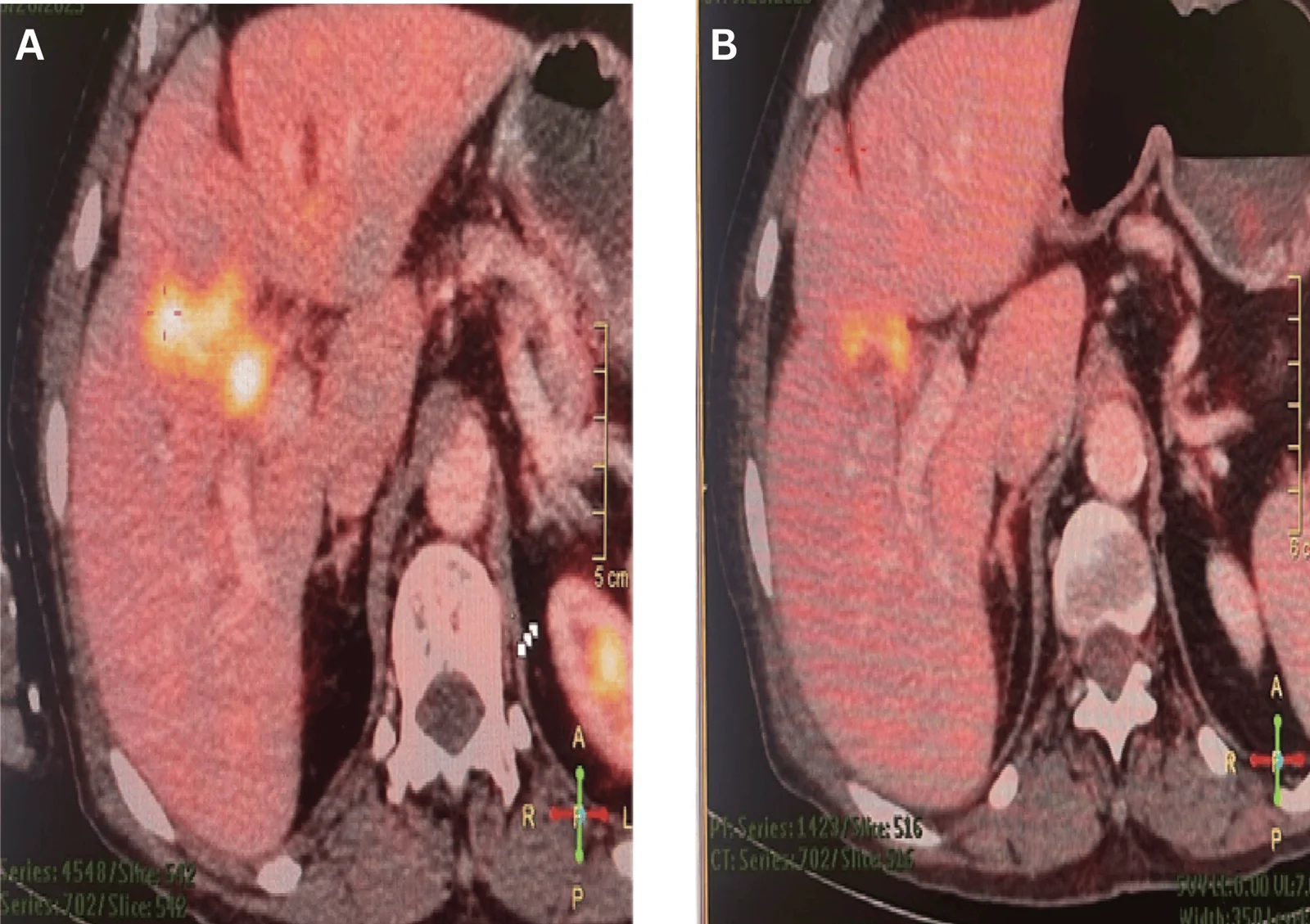
FDG PET-CT showing an FDG-avid, heterogeneously enhancing, infiltrative gallbladder mass, with a SUVmax of 8.3 at initial presentation (A). Follow-up imaging (B) demonstrates a reduced SUVmax of 6.3, supporting resolution of the inflammatory process associated with XGC FDG: fluorodeoxyglucose, PET-CT: positron emission tomography-computed tomography, SUVmax: maximum standardized uptake value, XGC: xanthogranulomatous cholecystitis

The initial and follow-up investigation results (after two months) are summarized in Table 1.

**Table 1:** Summary of investigations at initial presentation and at two-month follow-up demonstrating resolving inflammation in XGC AST: aspartate aminotransferase, ALT: alanine aminotransferase, SUVmax: maximum standardized uptake value, XGC: xanthogranulomatous cholecystitis

Investigations	Initial	Follow-up (after 2 months)	Normal range
Serum total bilirubin	9.10 mg/dL	1.2 mg/dL	0.1-1.2 mg/dL
Conjugated bilirubin	7.60 mg/dL	0.3 mg/dL	0.1-0.3 mg/dL
Serum AST	206 U/L	25 U/L	4-40 U/L
Serum ALT	276 U/L	16 U/L	4-41 U/L
Serum alkaline phosphatase	670 U/L	62 U/L	40-129 U/L
Gallbladder wall thickness (by ultrasound)	>10 mm	4 mm	≤3 mm
PET-CT SUVmax	SUVmax 8.3	SUVmax 6.3 (consistent with resolving inflammation)	-

Follow-up abdominal ultrasonography of the abdomen at two months showed a significantly reduced gallbladder wall thickness (4 mm). He returned to his usual state of health and maintains a good quality of life. The patient has subsequently remained under regular clinical surveillance with whole-abdomen ultrasonography and liver function tests. This ongoing surveillance is intended to monitor for any progressive gallbladder abnormalities and evidence of biliary obstruction, allowing reassessment if the clinical or radiological findings become concerning.

## Discussion

XGC is a rare variant of chronic cholecystitis that can closely resemble gallbladder carcinoma. The pathogenesis is believed to begin with cholelithiasis, with biliary obstruction increasing intraluminal gallbladder pressure, leading to rupture of Rokitansky-Aschoff sinuses and bile extravasation into the gallbladder wall, which triggers a xanthogranulomatous inflammatory response, causing fibrosis and extension into adjacent organs, mimicking neoplasm [2].

The differential diagnosis of a gallbladder mass includes gallbladder carcinoma, XGC, gallbladder empyema, gallbladder polyps, mucocele, and adenomyomatosis. The identification of a gallbladder mass in an elderly patient with abnormal liver function tests immediately raises concern for malignancy. In our case, the combination of advanced age, irregular gallbladder wall thickening, diffusion restriction and hepatic infiltration on MRCP, and intense FDG avidity on PET-CT initially supported a working diagnosis of gallbladder carcinoma. Acute suppurative processes, such as gallbladder empyema, were ruled out because the patient had no fever, no right upper quadrant pain, no leucocytosis, and no systemic signs of infection. Benign structural conditions such as adenomyomatosis typically demonstrate characteristic imaging findings including comet-tail artifacts on ultrasound and fluid-filled intramural diverticula/cysts on MRI. In our case, imaging did not demonstrate these hallmark features. A gallbladder polyp was ruled out by ultrasonography, which did not reveal discrete intraluminal polypoid lesions. Other inflammatory causes of wall thickening, such as uncomplicated chronic cholecystitis, were considered. However, the degree of FDG uptake and the apparent invasion into adjacent hepatic tissue were disproportionate to that typically seen in chronic inflammation. Because the patient presented with biliary obstruction, a primary biliary malignancy such as cholangiocarcinoma represents an important differential diagnosis. However, MRCP demonstrated that the dominant lesion was centered in the gallbladder, with the adjacent biliary obstruction attributable to compression by the gallbladder lesion rather than a discrete intrinsic common bile duct mass or primary ductal stricture. Similarly, metastatic deposits in the gallbladder were unlikely, as there was no evidence of a primary malignancy elsewhere in PET-CT. Thus, the principal diagnostic dilemma lay between gallbladder carcinoma and XGC. Radiologically, both entities can present with wall thickening, hepatic invasion, and FDG avidity. In our case, advanced age, irregular gallbladder wall thickening, dilated intrahepatic biliary radicles on MRCP, and extensive infiltration of the liver and surrounding structures favored gallbladder carcinoma. In contrast, the presence of a gallstone, intramural gallbladder collections, and the absence of local lymphadenopathy indicated a higher likelihood of XGC. We did not obtain tumor markers such as CA19-9, as previous studies have found that CA19-9 is unhelpful in differentiating benign from malignant gallbladder conditions, particularly in the setting of biliary obstruction and inflammation [4].

Radiologically, diffuse gallbladder wall thickening, continuous mucosal line enhancement, hypodense intramural nodules within the thickened wall, and cholelithiasis favor XGC, whereas discontinuous mucosal lines, focal mass formation, intrahepatic biliary dilatation, and extension into the liver and duodenum favor carcinoma [5]. However, they share several overlapping features that make differentiation challenging [6]. PET-CT, although valuable in staging malignancy, is particularly deceptive in XGC, as FDG avidity results from inflammatory activation and the expression of GLUT-1 and GLUT-3 receptors on macrophages rather than from true neoplastic metabolism [7]. Our patient’s PET-CT findings showing a heterogeneous FDG-avid lesion with hepatic infiltration exemplify this pitfall.

Histopathological confirmation remains the diagnostic cornerstone. The accuracy of EUS-FNB in distinguishing XGC from carcinoma has been reported to reach 96%, offering a minimally invasive and highly reliable diagnostic pathway [8]. However, a negative sample may not exclude carcinoma owing to sampling from non-representative areas. Intraoperatively, dense adhesions with surrounding structures may give the false impression of carcinoma. These findings can mislead surgeons to perform extended resections. In such scenarios, preoperative EUS-guided FNB and intraoperative frozen-section examination become indispensable for confirming the diagnosis. If an intraoperative frozen section confirms XGC, extended radical surgery may be avoided. Although XGC is benign, up to 20% of XGC cases have been associated with carcinoma, which may be easily overlooked due to sampling errors when both are present [9]. Therefore, follow-up imaging, such as PET-CT, is equally important for confirming the resolution of inflammation in XGC.

Cholecystectomy alone is the definitive treatment for XGC but is typically inadequate in the case of carcinoma, which requires radical resection of the gallbladder, liver, any involved viscera, and regional lymphadenectomy. Unnecessary extensive resections have been performed in XGC cases misdiagnosed as gallbladder carcinoma on preoperative imaging [10]. In frail elderly and comorbid patients, where surgical morbidity is substantial, EUS-guided tissue diagnosis and follow-up imaging are critical to avoid extensive resections like radical cholecystectomy. Integration of clinical, radiological, and histopathological data is essential for differentiating XGC from carcinoma and guiding appropriate management.

## Conclusions

XGC is a rare but clinically important inflammatory disease of the gallbladder that continues to pose a significant diagnostic challenge because of its striking resemblance to gallbladder carcinoma on clinical evaluation and imaging. Our case highlights how extensive gallbladder wall thickening, apparent hepatic infiltration, and intense FDG uptake on PET-CT created a strong radiological impression of malignancy. However, FDG avidity on PET-CT should not be taken as synonymous with malignancy, as inflammatory conditions such as XGC may exhibit similar metabolic activity, leading to false-positive interpretations. The diagnosis was ultimately established by histopathological examination, reaffirming that tissue confirmation remains the gold standard before committing patients to radical surgical procedures. In patients with imaging findings highly suggestive of gallbladder carcinoma, particularly when the clinical course is atypical or incongruent with advanced malignancy, alternative diagnoses such as XGC should remain an important consideration. Failure to recognize this possibility may expose patients to unnecessary radical resections, increased perioperative morbidity, and diminished quality of life. Although cholecystectomy remains the standard definitive treatment for XGC, management should be individualized according to the patient's clinical condition, operative risk, and treatment preferences. In select patients with XGC, particularly frail elderly individuals with significant comorbidities and high surgical morbidity, conservative management with close clinical surveillance and interval radiological assessment may represent a safe and effective strategy. A multidisciplinary approach integrating clinical assessment, high-quality imaging, histopathological confirmation, and thoughtful follow-up is essential to distinguish XGC from gallbladder carcinoma.
